# Screening and Characterization of *Pediococcus acidilactici* LC-9-1 toward Selection as a Potential Probiotic for Poultry with Antibacterial and Antioxidative Properties

**DOI:** 10.3390/antiox12020215

**Published:** 2023-01-17

**Authors:** Chong Li, Shaolong Wang, Si Chen, Xiaoying Wang, Xuejuan Deng, Guohua Liu, Wenhuan Chang, Yves Beckers, Huiyi Cai

**Affiliations:** 1 Key Laboratory for Feed Biotechnology of the Ministry of Agriculture and Rural Affairs, Institute of Feed Research, Chinese Academy of Agriculture Sciences, Beijing 100081, China; 2 Precision Livestock and Nutrition Laboratory, Teaching and Research Centre (TERRA), Gembloux Agro-Bio Tech, University of Liège, 5030 Gembloux, Belgium; 3 Department of Molecular Cell Biology, Samsung Medical Center, Sungkyunkwan University School of Medicine, Suwon 16419, Republic of Korea; 4 National Engineering Research Center of Biological Feed, Beijing 100081, China

**Keywords:** *Pediococcus acidilactici*, screening, antibacterial, antioxidant, broiler chickens, intestinal microbiota

## Abstract

Growing interest has been focused on lactic acid bacteria as alternatives to antimicrobial growth promoters, which are characterized by the production of various functional metabolites, such as antimicrobial and antioxidants compounds. The present study was undertaken to evaluate a potential probiotic from the antioxidant perspective. LC-9-1, screened from the intestines of healthy animals, was revealed to be *Pediococcus acidilactici* on the basis of its morphological, biochemical, and molecular characteristics. The strain has excellent properties, including acid-production efficiency, antibacterial performance and antioxidant activity. The safety of the strain was also evaluated. Furthermore, the experiments in broiler chickens suggested that dietary LC-9-1 supplementation improved the growth performance and decreased the abdominal fat, and enhanced the antioxidant capability and intestinal innate immunity of broilers. Analysis of intestinal microbiota showed that a higher community diversity (Shannon index) was achieved. In addition to the significantly increased relative abundances of *Pediococcus* spp., beneficial genera such as *Rothia* spp. and *Ruminococcus* spp. were abundant, while opportunistic pathogens such as *Escherichia-Shigella* spp. were significantly reduced in LC-9-1-supplemented broilers. Collectively, such in-depth characterization and the available data will guide future efforts to develop next-generation probiotics, and LC-9-1 could be considered a potential strain for further utilization in direct-fed microbial or starter culture for fermentation.

## 1. Introduction

Poultry meat is one of the most common animal-based food sources, and makes a significant contribution to world food security and human nutrition. However, poultry is susceptible to a variety of pathogenic microorganisms during intensive production [1]. In addition, oxidative stress is another major concern due to environmental heat stress, as well as pathological and nutritional factors, which also have a negative impact on the health and productivity of poultry [2].

Lactic acid bacteria (LABs) have been one of the most extensively investigated probiotics over several years [3]. Due to their being characterized by the production of functional metabolites, including antioxidants, organic acids and antibacterial compounds, they have been widely used in the fermentation industry and in animal production [4]. In recent years, foodborne infection and oxidative stress have become aggravated, and LABs have been designated as an important direct-fed microbiota (DFM) or probiotic starter culture [5]. Therefore, the further screening LAB with specific antibacterial and antioxidant capacities is of great significance.

In the present study, several LABs were screened and characterized from the intestinal tract of different healthy animals based on the antibacterial activity against selected pathogens and antioxidant capacity. Ultimately, only one strain was comprehensively assessed to determine its safety and efficacy in growing broilers.

## 2. Materials and Methods

### 2.1. Pathogenic Microorganisms

The pathogenic microorganisms of *Escherichia coli* (*E*. *coli*) ATCC25922, *Salmonella* ATCC13076 and *Staphylococcus aureus* (*S*. *aureus*) ATCC6538 were purchased from American Type Culture Collection (ATCC, Manassas, VA, USA), the *Proteus mirabilis* (*P. mirabilis*) CMCCB49005 was obtained from National Center for Medical Culture Collections (CMCC, Beijing, China). The microorganisms were cultured in nutrient broth (NB) medium, and routinely sub-cultured on NB agar (NBA) medium at 4 °C. All the mediums were purchased from Hope Bio-Technology Co., Ltd. (Qingdao, China).

### 2.2. Sample Collection and Isolation of LAB

The strains were isolated from the digestive tracts of healthy and free-ranging animals (the rumen of cattle, and the cecum of chickens, pigs and rabbits) without any additives during the rearing period (such as antibiotics or probiotics). All strains were collected in accordance with the Bioconvention and the Nagoya protocol [6]. LABs were isolated using the MRS-CaCO_3_ medium (Hope Bio-Technology Co., Ltd., Qingdao, China. CaCO_3_, 0.4%) according to the method described as Cho and Pan [7,8] with minor modification. A total of 237 colonies were categorized on the basis of their morphological characteristics and calcium-dissolving zone. The candidate strains were stored in MRS broth (Hope Bio-Technology Co., Ltd., Qingdao, China) with 20% glycerol at −80 °C.

### 2.3. Acid-Producing Efficiency (Primary Screen)

The method for evaluating LAB acidogenic efficiency was slightly modified from previous studies [9,10]. The selected strains were cultured at 37 °C for 18 h, and the inoculum was then introduced into the fermentation medium (100 g autoclaved peanut meal and 200 mL sterile brine with 40 g/L NaCl in a flask) at a rate of 4% (*v*/*v*). The cultures were incubated at 30 °C and 39 °C for 36 h under anaerobic conditions. The pH value was measured at the end of incubation using a pH-meter (FE20 pH meter, Changzhou, China). Each assay was performed in three independent tests and in triplicate.

### 2.4. Antibacterial Activity (Secondary Screen)

The antibacterial activity of LC-9-1 was determined by the standard agar well-diffusion method [5]. All assays were performed in triplicate.

### 2.5. Antioxidant Activity (Tertiary Screen)

The capacity of scavenging α-α-diphenyl-β-picrylhydrazyl (DPPH) radical of the candidate LAB was determined using the method explained by Lin et al. [11]. The Fenton reaction method was used for the assessment of hydroxyl radical scavenging test [12]. All tests were repeated three times.

### 2.6. Stress Tolerance (Tertiary Screen)

The stress tolerance of the candidate LAB was determined using the method reported by Kobierecka and Wang [13,14] with minor modifications. The survival of each LAB candidate was evaluated under different conditions. Briefly, the strains were grown overnight in MRS broth at 37 °C, and then transferred to fresh MRS broth (1:100 inoculum, *v*/*v*) within the following conditions for subculture: (a) adjusted to pH 6.2, pH 5.0, pH 4.0, pH 3.0 and pH 2.0 (with 1 M HCl), the control was broth without HCl, (b) adjusted to 0.1%, 0.2%, 0.3%, 0.4% and 0.5% (*w*/*v*) of bile salts (Sigma-Aldrich, St. Louis, MO, USA) with no pH adjustment, broth without bile salts was used as the control. All candidates were cultured at 37 °C for 4 h, the viable cells were counted by the plate count method and the results were expressed as survival rate. All tests were repeated four times.

### 2.7. Morphological, Biochemical, and Molecular Characteristics of LC-9-1

The characteristics of LC-9-1 were determined using the method explained by Bajpai et al. [15] with minor modifications. Firstly, the characteristic colonies were Gram stained, and bacteria were examined for morphology under the microscope (Olympus CX40, Olympus Optical Co. Ltd., Hamburg, Germany) and a scanning electron microscopic (SEM, Inspect F50, FEI Company, Hillsboro, OR, USA) [5]. A growth curve assay of LC-9-1 was performed with a previously described method [16]. 16S rRNA gene sequencing and phylogenetic analysis were adopted to specify the molecular characteristics of LC-9-1. Briefly, genomic DNA was isolated from LC-9-1 and then the 16S rRNA gene was amplified by PCR using the universal bacterial primers [17], 27F (5’-AGAGTTTGATCCTGGCTCAG-3’) and 1492R (5’-GGTTACCTTGTTACGACTT-3’) with the details of the procedure: 5 min at 95 °C for pre-deformation, 94 °C for 30 s, 57 °C for 30 s, 72 °C for 90 s for 30 cycles, and 72 °C for 8 min as the final step. The PCR products were sequenced by the Sangon Biotech Co., Ltd. (Shanghai, China). The homologies between the obtained gene sequences and those in GenBank were evaluated using BLAST analysis on the National Center for Biotechnology Information (NCBI). A bootstrap phylogenetic tree was constructed by the neighbor-joining method, using MEGA 7 software (www.megasoftware.net accessed on 15 March 2022). Furthermore, biochemical characteristics of LC-9-1 were performed using the API 50 CHL system (API 50 CHL, BioMerieux, Lyon, France) according to the instructions from the manufacturer, a standard strain of *Pediococcus acidilactici* ATCC 8042 (ATCC, Manassas, VA, USA) was used as a control in the test.

### 2.8. Safety Evaluation In Vitro of LC-9-1

#### 2.8.1. Hemolytic Activity Assay

The hemolytic activity of LC-9-1 was determined using the method explained by Maragkoudakis et al. [18] with minor modifications. Briefly, LC-9-1 was cultured in MRS broth overnight, and then inoculated in pre-made blood agar (Beijing Land Bridge Technology Co., Ltd., Beijing, China) containing 5% (*v*/*v*) sheep blood. The presence of a hemolysis zone was observed following incubation at 37 °C for 24 h.

#### 2.8.2. Antibiotic Susceptibility

Antibiotic susceptibility of LC-9-1 was determined using the agar diffusion method of CLSI [19] with minor modifications. Briefly, LC-9-1 was cultured in MRS broth at 37 °C for 18 h, and then preparing a suspension in accordance with two McFarland’s scales (10^8^ CFU/mL) [20]. A total of 100 μL suspension was spread onto MRS agar plate, followed by placement of antibiotic discs. The commercially antibiotic discs (HANGWEI, Hangzhou, China) contains Cefperazone (75 μg), Ceftriaxone (30 μg), Ceftazidime (30 μg), Cefuroxime (30 μg), Cefradine (30 μg), Cefazolin (30 μg), Cefalexin (30 μg), Minocyline (30 μg), Doxycycline (30 μg), Tetracycline (30 μg), Ciprofloxacin (5 μg), Clindamycin (2 μg), Erythromycin (15 μg), Neomycin (30 μg), Kanamycin (30 μg), Gentamicin (10 μg), Amikacin (30 μg), Vancomycin (30 μg), Piperacillin (100 μg), Ampicillin (100 μg), Oxacillin (1 μg), Penicillin (10 μg), Chloramphenicol (30 μg), Furazolidone (300 μg). Plates were incubated for 24 h at 37 °C and the diameters of the clear zones were measured and classified as sensitive (S), intermediate (I), and resistance (R) according to the guidelines for CLSI.

### 2.9. In Vivo Testing

#### 2.9.1. Strain Preparation

The *Pediococcus acidilactici* LC-9-1 was emulsified into microcapsules (prepared by Challenge Biotechnology Co., LTD (Beijing, China, viable count ≥5.0 × 10^10^ CFU/g). Following a conservative strategy, the amount of LC-9-1 in feed was examined daily throughout the experiment in order to ensure cell viability [21].

#### 2.9.2. Experimental Design and Bird Management

The trial was conducted at the Nankou pilot base of the Chinese Academy of Agricultural Sciences. The animal experiments were managed according to the National Institute of Animal Health approved protocol, and research reporting followed the guidelines of ARRIVE [22].

A total of 120 one-day-old male Arbor Acres (AA) broilers (body weight, 38.4 ± 0.7 g) were randomly allocated into 2 treatment groups with 6 replicates of 10 birds each. The control (CON) group was fed a basal diet, which met the nutritional requirements of broilers (Supplementary Table S1). The PA group was fed a basal diet containing 200 mg/kg LC-9-1 (5.5 × 10^9^ CFU/kg). The experiment lasted for 42 days in two feeding phases, starter (1–21 d) and grower (22–42 d), and all the broilers were housed in the same environmentally controlled facility (cleaning cage equipped with the fiberglass feeders and plastic net floor). All birds were allowed feed and water ad libitum, and given the same photoperiod (16 h light: 8 h dark), relative humidity (1–7 d, 60–70%; 8–42 d, 50–60%) and room temperature (1–7 d, 33 ± 2 °C; 8–16 d decreased stepwise to 24 °C; 17–42 d 24 °C). The excreta were cleared daily. Broilers were subjected a routine vaccination program, and their health was monitored daily.

#### 2.9.3. Sampling

Body weight (BW) and feed consumption were measured at 21 and 42 d of age, average daily gain (ADG), average daily feed intake (ADFI) and the feed intake/weight gain (F/G) ratio were calculated for the different phases. At 21 and 42 d of age, one broiler (close to the average BW) from each replicate was selected after a 12 h fasting. Blood samples (2.5 mL) were collected from the wing vein into EDTA-containing and anticoagulant-free vacuum test tubes (5 mL), respectively, and immediately placed on ice. Serum was harvested from nonanticoagulated whole blood by centrifuging at 3000× *g* for 10 min, and stored at −20 °C until analyzed. The slaughter performance and immune organ indexes were measured according to the production performance noun terms and metric statistics method of poultry (NY/T823-2004) [23,24] Liver, spleen and intestinal tissues (jejunum and ileum) were collected and fixed in 10% buffered formaldehyde (pH 7.4) for histological analysis. At 42 d of age, the ileal contents of 6 broilers were collected and snap-frozen in liquid nitrogen, followed by storage at −80 °C for DNA extraction. 

#### 2.9.4. Hematological and Serum Biochemical Indexes Analysis

The red blood cell count (RBC), hemoglobin concentration (HGB), white blood cell count (WBC), and lymphocytes (Lym) were measured with an auto hematology analyzer (XT-1800i, Sysmex Corporation, Tokyo, Japan). The alanine aminotransferase (ALT) and aspartate aminotransferase (AST) were measured using an automatic blood biochemical analyzer (AU640, Olympus Corporation, Tokyo, Japan). The total protein (TP) and albumin levels of serum were measured using commercial assay kits (Nanjing Jiancheng Bioengineering Institute, Nanjing, China) using a colorimetric method. The globulin content was obtained by subtracting the albumin value from that of the TP [25]. Total antioxidant capacity (T-AOC), activity of total superoxide dismutase (T-SOD), glutathione peroxidase (GSH-Px) and malonaldehyde (MDA) concentrations in the serum were determined by the commercial assay kits (Nanjing Jiancheng Bioengineering Institute, Nanjing, China) with an automated fluorescence instrument (MultiskanM™ SkyHigh, Thermo Fisher Scientific, Waltham, MA, USA).

#### 2.9.5. Histological Analysis

Fixed liver and spleen and intestine samples were dehydrated with increasing concentrations of ethyl alcohol (75%, 85%, 95%, and 100%), cleared in xylene, and embedded in paraffin. Five-micrometer-thick sections were prepared using a microtome, then the paraffin-embedded sections were deparaffinized, dehydrated and stained by hematoxylin–eosin (H&E). Visualization was performed under a light microscope (Olympus CX40, Olympus Optical Co. Ltd., Hamburg, Germany). For each section, tissue was observed at a magnification of ×40, gross lesions were observed, and the images were taken at magnifications of ×100 and ×400 (only for specific lesions). 

Epithelial thickness, villus length and crypt depth were measured at least 10 well-oriented villi, and the villus length/crypt depth ratio (V/C) ratio was calculated. Goblet cells were identified by periodic acid-Schiff (PAS) staining according to previously described method [26]. Target cells were stained purple and were counted as the number of cells per 100 μm of the villi.

#### 2.9.6. Microbial Analysis

Microbial genomic DNA of the intestinal contents was extracted under sterile conditions using the QIAamp DNA stool Mini Kit (Qiagen, Hilden, Germany) following the manufacturer’s instructions, and then the DNA integrity and purity were assessed by agarose gel electrophoresis and NanoDrop2000 (Thermo Fisher Scientific, Waltham, MA, USA), respectively. Subsequently, gene sequencing was implemented by Majorbio Biotech Co., Ltd. (Shanghai, China). The V3 to V4 variable region of the 16S rRNA gene was amplified with universal primers 338F and 806R. The PCR products were collected and sequenced using the Illumina MiSeq platform (Illumina, Madison, WI, USA). High-quality reads were filtered and clustered into operational taxonomic units (OTUs) based on sequences with ≥97% similarity and then analyzed using the QIIME software (version 1.9.1). The online platform (https://cloud.majorbio.com/ accessed on 3 June 2022.) of Majorbio Biotech Co., Ltd. was used to analyze the reads data. In particular, alpha-diversity indices including Chao1 index, Shannon index, Coverage index and numbers of OUTs were analyzed by Student’s *t*-test at OTU level. The beta-diversity analysis includes the principal component analysis (PCA) and principal coordinate analysis (PCoA) [27]. The Kruskal–Wallis rank sum test was employed for analysis of the relative abundance at the phylum and genus levels. Linear discriminant analysis (LDA) effect size (LEfSe) analysis was implemented using the non-parametric factoria Kruskal–Wallis rank sum test to obtain significantly different species between the CON and PA group [28], differences between groups were assessed using the Wilcoxon rank sum test, and finally LDA was used to access the influence of each species abundance on the differences.

### 2.10. Statistical Analysis

The data were analyzed by a one-factor ANOVA procedure using SPSS 19.0 software package for Windows (SPSS Inc., Chicago, IL, USA). Significant differences between groups were separated using Duncan’s multiple range test. Differences were considered significant at *p* < 0.05. The graphs were designed using GraphPad Prism 9 Project (GraphPad Software Inc., San Diego, CA, USA) and Origin 8.5 (Origin Lab, Berkeley, CA, USA).

## 3. Results

**Figure 1:**
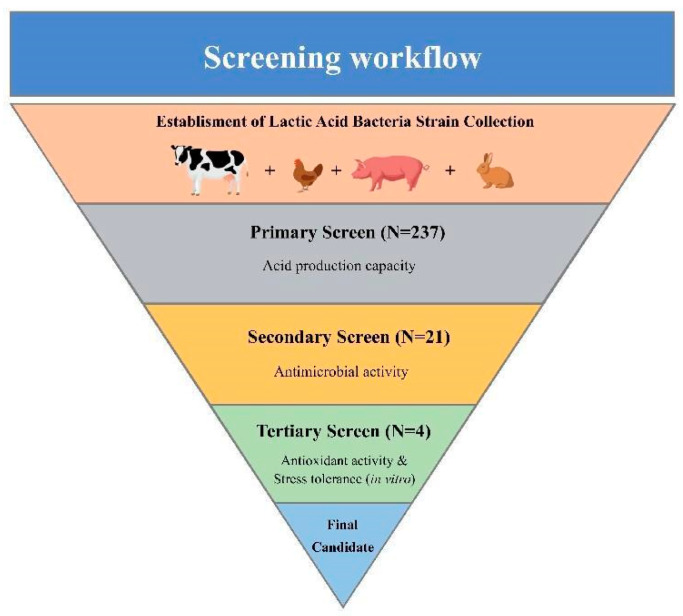
Workflow of the screening. The process was divided into three parts that were performed to narrow down the total of 237 potential LAB isolates to the one strain with the highest observed antibacterial and antioxidative activity.

A total of 237 potential LAB isolates were isolated, and the workflow is given in Figure 1.

### 3.1. Primary Screen

In the primary screen, potential strains with strong acid-producing capacity were selected by culturing at 30 °C and 39 °C. The best 30 isolates in terms of their acid-producing potential are shown in Supplementary Figure S1, of which 21 strains appeared in both datasets and were further characterized.

### 3.2. Secondary Screen

After primary screening, the most promising strains of LAB were tested for antimicrobial activity against four pathogenic microorganisms. The well-diffusion test resulted in the isolation of 10 strains with the strongest inhibitory effects of different pathogens (Supplementary Figure S2), among which four isolates (LC-2-5, LC-2-9, LC-3-9 and LC-9-1) were found to be the most prevalent strains and were selected for further analysis.

### 3.3. Tertiary Screen

**Figure 2:**
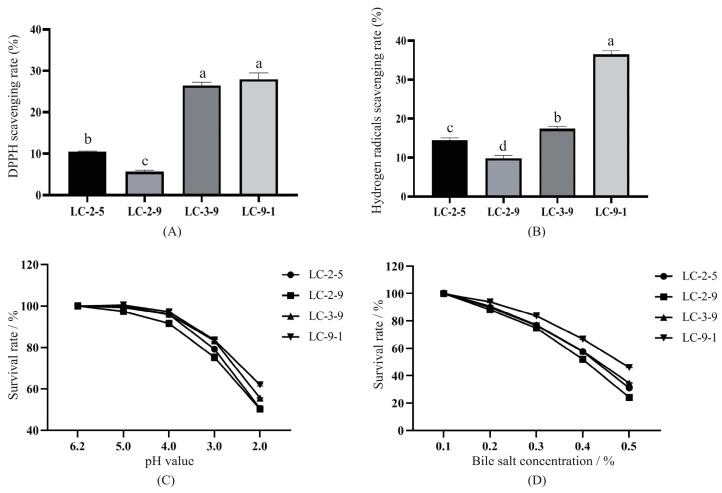
Antioxidant activity and stress tolerance of the candidate LAB. (**A**) DPPH scavenging activity; (**B**) hydroxyl radical scavenging activity; (**C**) tolerance of the strains to different pH treatments; (**D**) tolerance of the strains to different concentrations of bile salts.

As shown in Figure 2A, the scavenging effects of candidates on DPPH radicals ranged from 5.66% to 27.99%. LC-9-1 and LC-3-9 exhibited the highest removal efficiency, with no significant difference between the two strains. Figure 2B shows the hydroxyl radical scavenging activity of the four strains, which ranged from 9.82% to 36.42%. The efficiency of LC-9-1 was significantly higher than that of the other strains, with a scavenging activity of 36.42% (*p* < 0.05).

The tolerance of the strains to acid/alkaline conditions is shown in Figure 2C, with almost all of them demonstrating proliferation inhibition at pH below 5. The survival rates of the strains were significantly different after being treated at pH 2.0 (*p* < 0.05). LC-9-1 had the highest survival rate of 61.96%, followed by LC-3-9, the lowest were LC-2-5 and LC-2-9. The results of tolerance to bile salts are showed in Figure 2D. Overall, the survival rates of the four hit strains were similar when treated with 0.1% and 0.2% level bile salts, then declined with bile salt level. The survival rates of LC-3-9, LC-2-5 and LC-2-9 were all below 40% under 0.5% bile salt conditions, while the most tolerant stain was LC-9-1, with a survival rate of 46.01%; thus, it was chosen for the subsequent trials.

### 3.4. Morphological, Biochemical, and Molecular Characteristics of LC-9-1

**Figure 3:**
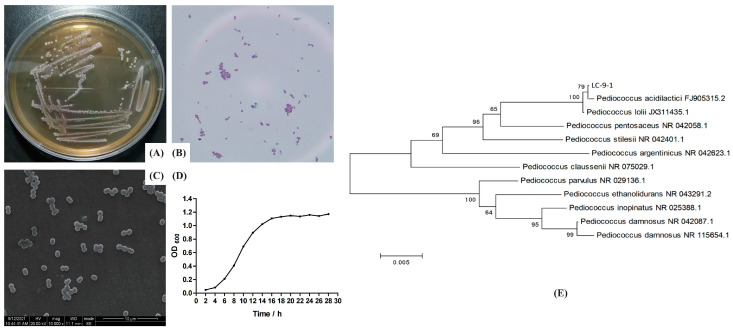
Morphological, biochemical, and molecular characteristics of LC-9-1. (**A**) Colony morphology; (**B**) Gram stain showing Gram-positive cocci; (**C**) SEM image of LC-9-1 showing a spherical morphology (×1000); (**D**) growth curve of LC-9-1; (**E**) phylogenetic tree analysis of LC-9-1.

The isolate of LC-9-1 appeared as creamy white, opaque, round and small colonies on the surface of MRS agar of the isolate LC-9-1, which was confirmed to be Gram-positive cocci (spherical shaped) by microscopic evaluation (Figure 3A,B). The cells tended to occur in pairs (Figure 3C). The proliferation curves appeared in a typically sigmoidal shape, consisting of a latency phase (0–4 h), a logarithmic phase (4–16 h) and a plateau phase (16 h later) (Figure 3D). According to the 16S rRNA gene sequencing and phylogenetic characteristics, the similarity of the LC-9-1 strain to *Pediococcus* spp. was 99.9% (Figure 3E); thus, the strain was preliminarily identified as *P. acidilactici*. Biochemical analysis was performed using the API 50 CHL strip kit and the strip capsules turned yellow, indicating a complete fermentation of sugar by strain. The API web software confirmed that LC-9-1 utilized carbohydrates including Salicin, D-cellobiose, D-ribose, D-xylose, D-saccharose, D-trehalose, D-galactose, D-glucose, D-fructose, D-mannose, Gentiobiose, D-tagatose, N-acetylglucosamine, Amygdalin, Arbutin and Esculin (Supplementary Table S2). The strain exhibited no difference in color change compared to the standard strain of *P. acidilactici* ATCC 8042, suggesting that both strains had the same fermentation of sugar pathways. Taken together, LC-9-1 was identified as *P. acidilactici* and has been deposited in the China General Microbiological Culture Collection Center (CGMCC, Beijing, China), with the patent number (CGMCC No. 21345).

### 3.5. Safety Evaluation In Vitro of LC-9-1

The hemolytic activity of LC-9-1 was judged by observing the hemolytic rings on blood agar plates after a 24 h incubation. The strain was not involved in the lysis of erythrocytes (results not shown). Supplementary Table S3 shows the antibiotic susceptibility profile of the LC-9-1, validating that it was susceptible to most antibiotics. Specifically, the strain was extremely susceptible to Cefoperazone, Ceftriaxone, Erythromycin, Cefuroxime, Cefradine, Cefalexin, Minocyline, Doxycycline, Tetracycline, Piperacillin, Ampicillin, Penicillin, Chloramphenicol, Furazolidone and Clindamycin. Additionally, it was found that LC-9-1 was completely resistant to Kanamycin, Gentamicin, Amikacin and Ciprofloxacin.

### 3.6. In Vivo Testing

#### 3.6.1. Growth Performance

**Figure 4:**
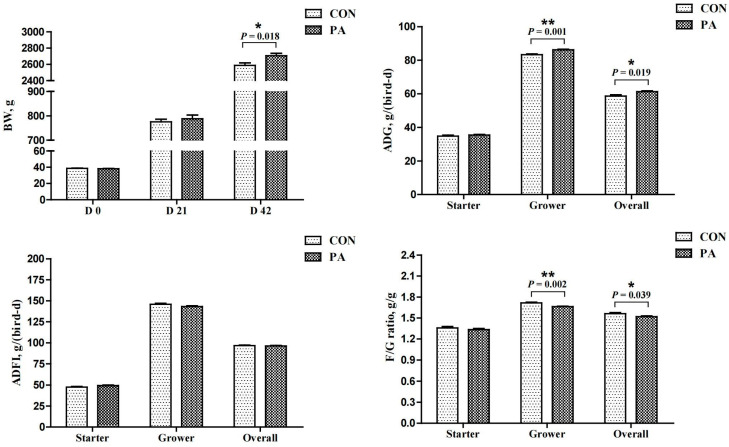
Effect of *P. acidilactici* LC-9-1 on growth performance of broilers (*n* = 6). CON = control group, broilers were fed a corn–soybean basal diet, PA = *P. acidilactici* group, broilers were fed a basal diet containing 5.5 × 10^9^ CFU/kg *P. acidilactici* LC-9-1. BW, body weight; ADG, average daily gain; ADFI, average daily feed intake; F/G ratio = feed intake (g)/weight gain (g). The values with superscript (*) were significantly different, * 0.01 < *p* < 0.05; ** *p* ≤ 0.01.

The average mortality rate was 0.5% during the experiment (data not presented), and there were no significant differences between the groups. The response of growth performance is shown in Figure 4. Compared with the CON group, LC-9-1 significantly increased the BW of broilers at 42 d of age and the ADG during the grower and overall periods (*p* < 0.05), and decreased the F/G ratio during the grower and overall periods (*p* < 0.05). No significant differences were found in the ADFI.

#### 3.6.2. Slaughter Performance and Immune Organ Indexes

The effect of LC-9-1 on slaughter performance of broilers is listed in Table 1. Dietary supplementation with LC-9-1 did not have a negative effect on the relevant parameters. However, the abdominal fat rate of broilers in PA group was significantly lower than that in CON group (*p* < 0.01). In addition, no effect was observed on immune organ indexes of broilers at 21 and 42 d of age (Table 2).

#### 3.6.3. Hematological and Serum Biochemical Index Analysis

As shown in Table 3, compared with the CON group, LC-9-1 treatment did not affect the hematological or serum biochemical indexes of birds at 21 d of age. However, at 42 d of age, LC-9-1 treatment significantly decreased ALT and AST and elevated A/G ratio compared to the CON group (*p* < 0.05).

#### 3.6.4. Serum Antioxidant Performance

The effect of LC-9-1 on serum antioxidant of broilers is shown in Table 4. Feeding with LC-9-1 significantly increased the activities of T-AOC and T-SOD at 21 and 42 d of age compared to the basal diet (*p* < 0.05). Other than that, LC-9-1 supplementation significantly decreased the MDA concentration at 21 d of age (*p* < 0.05). Nevertheless, no significant difference was found between the two groups regarding the GSH-Px activity at 21 and 42 d of age.

#### 3.6.5. Histological Analysis

**Figure 5:**
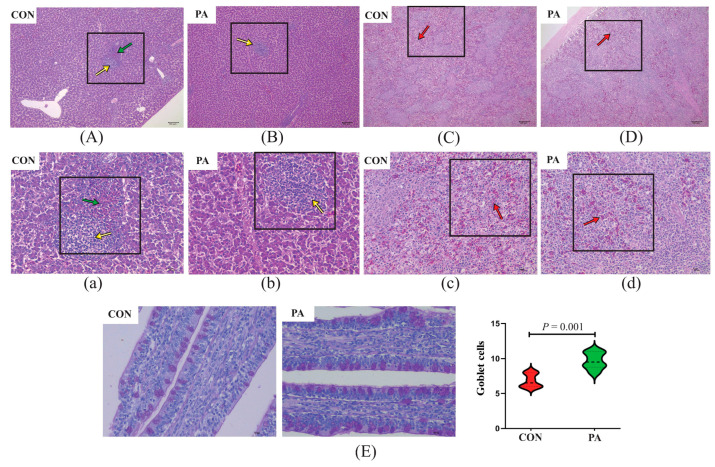
Effect of *P. acidilactici* LC-9-1 on histological analysis of organs and ileal epithelial goblet cells in broilers at 21 a of age (*n* = 6). CON = control group, broilers were fed a corn–soybean basal diet, PA = *P. acidilactici* group, broilers were fed a basal diet containing 5.5 × 10^9^ CFU/kg *P. acidilactici* LC-9-1. (**A**,**B**) Liver H&E stained (100×); (**C**,**D**) spleen H&E stained (100×); (**a**,**b**) liver H&E stained (400×); (**c**,**d**) spleen H&E stained (400×). Yellow arrowheads indicate lymphocytic aggregates, green arrowheads indicate heterophilic granulocyte, red arrowheads indicate splenic congestion. (**E**) Representative images of ileal PAS stained (400×) and statistical analysis of the number of epithelial goblet cells.

**Figure 6:**
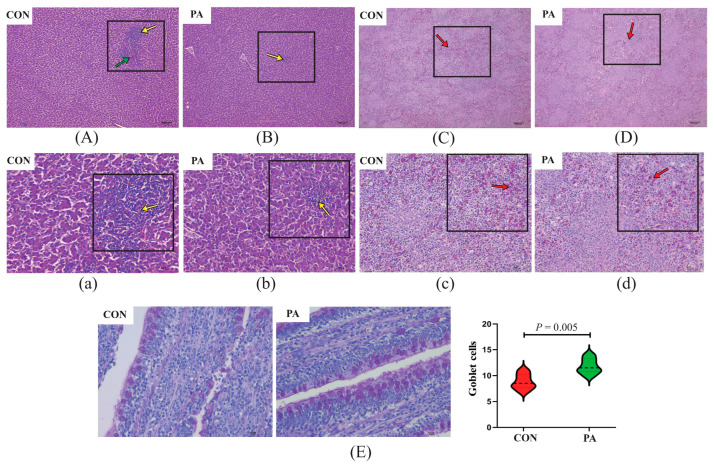
Effect of *P. acidilactici* LC-9-1 on histological analysis of organs and ileal epithelial goblet cells in broilers at 42 d of age (*n* = 6). CON = control group, broilers were fed a corn–soybean basal diet, PA = *P. acidilactici* group, broilers were fed a basal diet containing 5.5 × 10^9^ CFU/kg *P. acidilactici* LC-9-1. (**A**,**B**) Liver H&E stained (100×); (**C**,**D**) spleen H&E stained (100×); (**a**,**b**) liver H&E stained (400×); (**c**,**d**) spleen H&E stained (400×). Yellow arrowheads indicate lymphocytic aggregates, green arrowheads indicate heterophilic granulocyte, red arrowheads indicate splenic congestion. (**E**) Representative images of ileal PAS stained (400×) and statistical analysis of the number of epithelial goblet cells.

Histological examination of livers and spleens were mainly focused on the presence and extent of regressive lesions, such as lymphocytic aggregates, heterophils exudate, congestion and necrosis of the liver and spleen parenchyma. As shown in Figure 5 and Figure 6, histological examination of the liver and spleen did not reveal abnormal pathological lesions in the LC-9-1-treated broilers. Concretely, at 21 and 42 d of age, the CON group exhibited occasional lymphocytic aggregated and inflammatory infiltrates in the liver tissues, while only small-range lymphocyte aggregates were observed in the PA group. Additionally, the congestion and lymphocyte infiltration in the spleen tissues were rare in the LC-9-1 group, but more common in the CON group at 21 and 42 d of age.

The effect of LC-9-1 on the intestinal histomorphology of broilers is shown in Table 5. At 21 d of age, there were no significant differences in the histomorphometric features of the jejunum and ileum between the CON and PA groups. At 42 d of age, the villus length and V/C ratio in the ileum were significantly higher in the PA group than in the CON group (*p* < 0.05). However, LC-9-1 did not affect the epithelial thickness of jejunum and ileum at 21 and 42 d of age. The number of goblet cells in the ileal villus were significantly increased in the LC-9-1 group at 21 and 42 d of age (*p* < 0.05, Figure 5E and Figure 6E).

#### 3.6.6. Microbial Analysis

**Figure 7:**
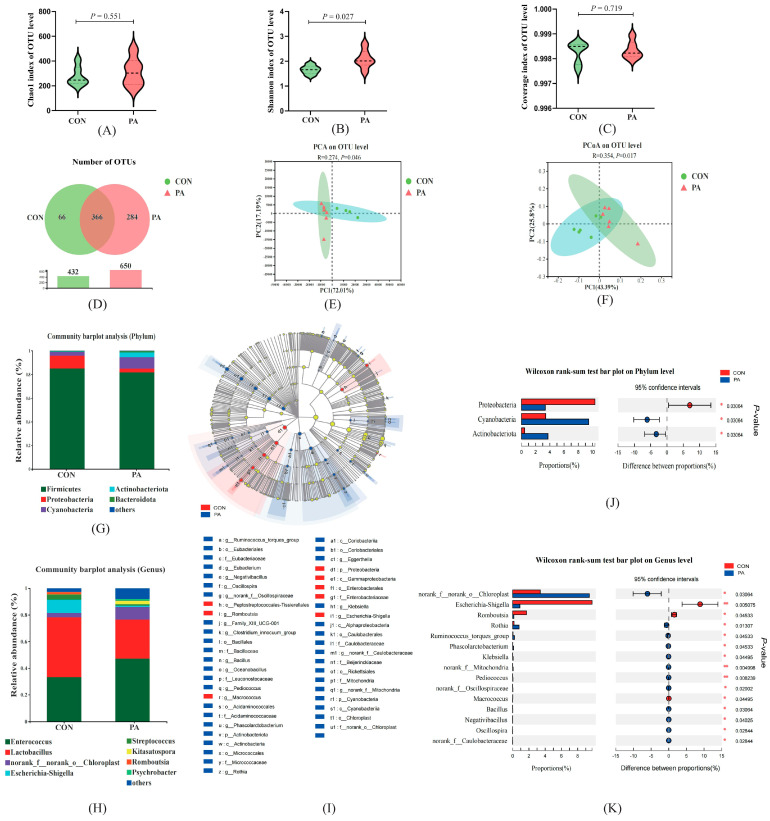
Effect of *P. acidilactici* LC-9-1 on gut microbiota in broilers at 42 d of age (*n* = 6). (**A**) Chao1 index of OUT level; (**B**) Shannon index of OUT level; (**C**) coverage index of OUT level; (**D**) number of OTUs; E&F, β-diversity was estimated by the PCA (**E**) and PCOA (**F**) on OUT level; (**G**,**H**) relative abundance of bacteria, (**G**) at the phylumand level and (**H**) at genus level; (**I**) cladogram of LEfSe multi-level species difference discriminant analysis (LDA > 2), different-color nodes indicate microbial communities that are significantly enriched in the corresponding groups and significantly different between groups; (**J**,**K**) comparative analysis of the relative abundance of bacteria, (**J**) at the phylum level and (**K**) at genus level.

To understand whether LC-9-1 could reshape the gut microbiota community, we investigated the change in the ileal microbiota composition (Figure 7). The results revealed that the Chao1 index of OTU level was not significantly different between the groups (Figure 7A), while the Shannon index of OTU level was significantly higher in the PA broilers (*p* < 0.05) (Figure 7B), indicating the treatment of LC-9-1 caused a certain degree of change in the intestinal microbiota diversity. The coverage index for every sample was greater than 0.997 (Figure 7C), indicating that the subsequent analyses were not affected by biases in sequencing depth. Furthermore, an increasing trend in the number of OTUs was observed in the PA group compared with the CON group (Figure 7D). The *β*-diversity analysis is shown in Figure 7E,F, and the results of PCA and PCoA showed that samples in the CON and PA groups had different community compositions and structures, suggesting that a significant segregation had occurred between microbiota groups. The stacked bar graphs were created to show the different OTUs at the level of Phylum and Genus (Figure 7G,H). Regarding the results indicating the quantity of OTUs at the phylum level, five bacteria had an average relative abundance >1% for each group, of which the most abundant in the CON group were Firmicutes and Proteobacteria, while in the PA group, the most abundant were Firmicutes and Cyanobacteria. At the genus level, a total number of eight bacteria had relative abundances above 1%, of which the most abundant in the CON were *Lactobacillus*, *Enterococcus* and *Escherichia-Shigella*, while in the PA group the most abundant were *Enterococcus*, *Lactobacillus* and *Chloroplast*. Additionally, LEfSe analysis (LDA > 2) revealed significant differences in microbiota structure between the CON group and the PA group (Figure 7I). Furthermore, adopting the Kruskal–Wallis rank sum test, we found that Cyanobacteria and Actinobacteriota at the Phylum level were more abundant (*p* < 0.05) in the PA group than in the CON group (Figure 7J), while the abundance of Proteobacteria was significantly decreased (*p* < 0.05). At the genus level (Figure 7K), 15 bacteria were significantly different, the relative abundance of *Escherichia-Shigella, Romboutsia* and *Macrococcus* were notably decreased in the PA group (*p* < 0.05), while other species of bacteria such as *Chloroplast*, *Rothia* and *Ruminococcus* were significantly elevated (*p* < 0.05). Importantly, we also found a significant increase in the relative abundance of *Pediococcus* in the PA group (*p* < 0.01).

## 4. Discussion

LABs are recognized as an excellent probiotic that produces organic acids, bacteriocin, and also have excellent antioxidative properties [11,29]. The bacteria decompose carbohydrates into organic acids during fermentation, which play an important role in improving the nutrition and storage quality of food [30]. In addition, it has been shown that organic acids could improve the innate immunity of poultry [31]. Based on this, the acid production efficiency of LAB was used as an evaluation index in the primary screen. The antibacterial activity of LAB was determined by secreting different antibacterial substances, including antibacterial peptides, bacteriocins, hydrogen peroxide, alcohol, organic acids, etc. According to Reuben’s report, organic acids and small molecular substances were the most promising components [20], which is another factor recommending acid-producing efficiency as the primary screening criterion in our study. Furthermore, the antibacterial effects of screened LABs were tested against *E. coli*, *Salmonella*, *S. aureus* and *P. mirabilis*, which are the most common pathogenic species in poultry infections [32,33,34,35]. The most widely used commercial rearing system at present is closely related to oxidative stress in broilers [36]. Serious oxidative stress can lead to inferior growth performance, tissues damage and even death [37]. A variety of LAB have been shown to have antioxidant activity, and antioxidant enzymes have been proven to be important defense systems against oxidative stress, such as lipoteichoic acid, exopolysaccharides and cell-surface proteins [11]. In tertiary screening, the scavenging ability of LAB on DPPH free radicals and hydroxyl radical were taken as an evaluation index, and the performance of potential probiotics in terms of acid resistance and bile salt tolerance is also important for their survival and growth in the intestinal tract [38]. Finally, the LC-9-1 strain showed the best performance among the candidates. According to its morphological, biochemical and molecular characteristics, we finally identified the target strain as *P. acidilactici*. Although the majority of LAB are generally recognized as safe [39], a safety evaluation of LC-9-1 was also undertaken. The strain was not involved in the lysis of erythrocytes, and it was validated as being susceptible to most antibiotics. Furthermore, LC-9-1 was found to be resistant to aminoglycoside antibiotics (kanamycin, gentamicin, amikacin) and quinolone antibiotics (ciprofloxacin). It is worth noting that many species of LAB are intrinsically resistant to antibiotics, such as kanamycin, gentamicin, vancomycin, ciprofloxacin and streptomycin, but the antibiotic resistance genes could not be horizontally transmitted between different bacterial communities [14,40]. These results illustrate that LC-9-1 has potential as a safe probiotic candidate.

In vitro assessment may not be representative of the situation in vivo; therefore, we evaluated the safety and application potential of the LC-9-1 by adding it to broiler diets. The results showed that 5.5 × 10^9^ CFU/kg of LC-9-1 increased the body weight at 42 d of age and increased the ADG during the grower and overall periods of broilers. Previous studies have shown that *P. acidilactici* combined with Xylan oligosaccharides and mannan-oligosaccharides, respectively, could benefit the intestinal health and growth performance of broilers, through possible mechanisms including improving intestinal morphology, optimizing the intestinal microflora structure, and reducing the relative abundance of pathogenic microorganisms [41,42]. Moreover, the data showed that dietary LC-9-1 supplementation not only had no negative effects on slaughter performance and immune organ indexes, but also significantly reduced abdominal fat in broilers. With the constant improvement in the growth speed of broilers, the problem of excessive abdominal fat deposition is becoming more prominent. It has been reported that excessive fat is associated with decreased immunity and the occurrence of various diseases [43]. Excessive abdominal fat not only reduces the immunity of broilers to diseases, it also reduces the economic value of the broilers. Therefore, reducing broilers’ abdominal fat deposition has become one of the main tasks for researchers in the broiler industry.

Broilers are often affected by various stressors under the conditions of intensified and industrialized modes of production, which directly or indirectly cause tissue damage, including in the intestinal tract and liver. The activity levels of serum ALT and AST are considered to be diagnostic tools for liver injury, and any pathological or toxic injury may result in elevated activity levels of ALT and AST [44]. It was found in the present study that LC-9-1 reduced the activity levels of ALT and AST, indicating that the liver injury was recovered to a certain extent. This result was also verified by liver histological examination; the lesions of the PA broilers were milder than those of the CON broilers, indicating that LC-9-1 had a certain protective effect on the liver. There is a point that requires clarification. It has previously been shown that cage-rearing systems can induce an increase in serum MDA, which leads to a higher risk of tissue damage [45,46]. We consider that the CON broilers showed tissue damage that was associated with the cage rearing system applied. Moreover, only a small range lymphocyte aggregates were observed in the LC-9-1 broilers, which may be related to the antioxidant effect of LC-9-1.

Albumin level is associated with systemic inflammatory response and reflects nutritional status, and globulin level is associated with chronic inflammation [47,48]. Furthermore, the decrease in A/G ratio indicated an inflammation in poultry [49]. In this study, LC-9-1 significantly increased the A/G ratio compared with the CON group, and we speculated that LC-9-1 protects the physiological function of the liver and maintains the liver’s supplement of serum albumin. Moreover, LC-9-1 also reduces the inflammation level of the body and maintains a normal level of globulin.

As an adaptive mechanism, broilers tend to undergo physiological changes in stressful environments. High-density feeding not only induces oxidative stress, it also impairs the immune and antioxidant systems [50]. In biological systems, oxidative stress is usually caused by an imbalance between antioxidant and pro-oxidative system. Excessive generation of reactive oxygen species (ROS) causes oxidative damage to cells, and then triggers biological damage, impairing growth [51,52]. T-AOC reflects the ability of the non-enzymatic antioxidant defense system [53]. T-SOD regulates the balance of oxidation and anti-oxidation in vivo through enzymatic reactions, and it is seen as the first line of the antioxidant system by neutralizes the most unstable ROS superoxide anion to hydrogen peroxide. MDA is an end product of lipid peroxidation and is used as a biomarker of oxidative stress [54]. In the present study, the increased activities of T-AOC and T-SOD, and the decreased MDA concentration in the PA group indicated that the LC-9-1 improves the potential of the broilers’ endogenous antioxidant defense capacity. The mechanism for this may be related to the antioxidant properties of LC-9-1. Similar to our results, Lin et al. reported that supplementation with *L. plantarum* AR501 significantly increased the T-AOC activities in the mice [11]. It is well known that inflammation is one of the major downstream responses to oxidative stress, and inflammation can further aggravate oxidative stress [55]; this connection is increasingly evident in biological processes and cannot be ignored. For the above reasons, the resistance to pathogenic microorganisms and antioxidant activity of LAB were taken as screening principles in this study.

The absorption site of nutrients is mainly in the small intestine in broilers. The height of the villus indicates the area of intestinal absorption [56]. The V/C ratio represents the functional state of the intestine [57]. Probiotics have been demonstrated to protect the integrity of the gut tissue through various mechanisms [58,59]. Similar results were observed in our study, with LC-9-1 being beneficial to maintaining the morphology of the intestinal epithelium, and this provided a reasonable explanation for the improvement of growth performance in the PA group. Mucin secreted by goblet cells was primarily involved in innate host defense [60]. In this study, the intestinal goblet cells of broilers in the PA group were significantly increased at 21 and 42 d of age, indicating that LC-9-1 may enhance intestinal innate immunity.

The intestinal microbiota community plays a variety of roles in nutrient absorption and metabolism, immunity, and host health [61]. It has previously been shown that the diversity of gut microbes contributes to microbiome homeostasis and the resistance to pathogenic microorganisms [62]. In this study, dietary supplementation with LC-9-1 increased the intestinal microbial diversity (Shannon index), but did not significantly alter the microbial community richness (Chao1 index). The PCA and PCoA analyses showed significant differences in microbial communities between the PA group and CON group. The predominant bacterial phyla in the ileum included Firmicutes, Cyanobacteria, Proteobacteria and Bacteroidetes [63], similar to our observations. The most abundant phyla in the CON broilers were Firmicutes and Proteobacteria, while in the PA group the most abundant were Firmicutes and Cyanobacteria. Proteobacteria contains a wide variety of pathogens, such as *Salmonella*, *Escherichia coli*, and *Shigella*, which could exert pathogenic effects in the intestine of chickens [64]. The decrease in Proteobacteria indicated that a relatively healthy bacterial community was achieved with LC-9-1 supplementation. Furthermore, analysis at the genus level verified the above issue. Supplementation with LC-9-1 reduced the relative abundance of *Escherichia-Shigella*, which is an opportunistic pathogen and is positively correlated with a variety of intestinal infections [65]. We also found a similar reduction in *Romboutsia*, which is associated with *C. difficile* infection [66]. Correspondingly, we found an increase in the abundance of some beneficial genera, such as *Rothia* and *Ruminococcus*. Most importantly, the supplemented LC-9-1 belongs to genus *Pediococcus,* which also had significantly higher relative abundance in PA broilers. Taken together, it can be concluded that LC-9-1 contributes to maintaining the microbial balance in the intestine, and promoting the growth of beneficial bacteria, while suppressing potentially pathogenic microbes.

## 5. Conclusions

This study showed the newly screened and characterized LAB *P. acidilactici* LC-9-1, which was isolated from the intestinal tract of different healthy animals. Specifically, the *P. acidilactici* LC-9-1 had excellent acid-producing ability, antibacterial properties, antioxidant ability and stress resistance in vitro. The in vivo experiments indicated that dietary LC-9-1 supplementation improved the growth performance, reduced the abdominal fat, enhanced the antioxidant capacity, and improved the innate immunity level of intestinal tract. The mechanism may be related to the various functional metabolites produced by LC-9-1 and its influence on the intestinal microbiota. Therefore, *P. acidilactici* LC-9-1 could be considered a potential strain for further utilization in DFM or probiotic starter culture.

## Figures and Tables

**Table 1:** Effect of *P. acidilactici* LC-9-1 on slaughter performance of broilers at day 42 (*n* = 6).

Items	CON	PA	SEM	*p*-Value
Dressing percentage, %	89.55	90.53	0.471	0.320
Half-eviscerated yield, %	84.86	86.30	0.457	0.117
Eviscerated yield, %	75.13	75.63	0.484	0.628
Breast muscle ratio, %	13.67	13.63	0.231	0.963
Thigh muscle ratio, %	10.99	10.93	0.353	0.934
Abdominal fat ratio, %	2.22 **	1.76	0.083	0.001

CON = control group, broilers were fed a corn–soybean basal diet, PA = *P. acidilactici* group, broilers were fed a basal diet containing 5.5 × 10^9^ CFU/kg *P. acidilactici* LC-9-1. SEM = standard error of means. The values with superscript (*) in the same row were significantly different, ** *p* ≤ 0.01.

**Table 2:** Effect of *P. acidilactici* LC-9-1 on immune organ indexes of broilers (*n* = 6).

Items	Dietary Treatment		SEM	*p*-Value
	CON	PA		
Day 21				
Spleen index, mg/g	0.88	0.99	0.040	0.198
Thymus index, mg/g	2.85	2.84	0.017	0.784
Bursa of fabricius index, mg/g	1.96	1.98	0.011	0.387
Day 42				
Spleen index, mg/g	1.17	1.22	0.016	0.111
Thymus index, mg/g	2.17	2.19	0.013	0.397
Bursa of fabricius index, mg/g	1.04	1.04	0.019	0.901

CON = control group, broilers were fed a corn–soybean basal diet, PA = *P. acidilactici* group, broilers were fed a basal diet containing 5.5 × 10^9^ CFU/kg *P. acidilactici* LC-9-1. Spleen index, thymus index and bursa of fabricius index were calculated as follows: (1) spleen index (mg/g) = spleen weight (mg)/body weight (mg), (2) thymus index (mg/g) = thymus weight (mg)/body weight (g), (3) bursa of fabricius index (mg/g) = bursa of fabricius (mg)/body weight (g); SEM = standard error of means.

**Table 3:** Effect of *P. acidilactici* LC-9-1 on hematological and serum biochemical indexes of broilers (*n* = 6).

Items	Dietary Treatment		SEM	*p*-Value
	CON	PA		
Day 21				
RBC, ×10^12^/L	2.42	2.48	0.051	0.569
HGB, g/L	100.83	103.67	2.041	0.592
WBC, ×10^9^/L	144.90	140.95	1.290	0.130
LYM, ×10^9^/L	52.81	50.01	1.270	0.292
ALT, U/L	2.22	2.10	0.031	0.054
AST, U/L	239.73	230.36	2.465	0.051
TP, g/L	3.17	3.21	0.024	0.377
Albumin, g/L	1.38	1.41	0.013	0.248
Globulin, g/L	1.79	1.80	0.013	0.636
A/G ratio	0.77	0.79	0.005	0.286
Day 42				
RBC, ×10^12^/L	2.62	2.44	0.054	0.112
HGB, g/L	105.17	105.33	2.136	0.971
WBC, ×10^9^/L	143.99	140.17	1.228	0.123
LYM, ×10^9^/L	56.92	56.74	0.831	0.920
ALT, U/L	3.22 **	3.00	0.038	0.001
AST, U/L	244.10 **	228.01	3.040	0.002
TP, g/L	3.04	3.01	0.027	0.628
Albumin, g/L	1.30	1.33	0.016	0.257
Globulin, g/L	1.74	1.67	0.018	0.068
A/G ratio	0.75	0.80 **	0.010	0.003

CON = control group, broilers were fed a corn–soybean basal diet, PA = *P. acidilactici* group, broilers were fed a basal diet containing 5.5 × 10^9^ CFU/kg *P. acidilactici* LC-9-1. RBC, red blood cell count; HGB, hemoglobin concentration; WBC, white blood cell count; Lym, lymphocytes; ALT, alanine aminotransferase; AST, aspartate aminotransferase; TP, total protein; A/G ratio = albumin/globulin; SEM = standard error of means. The values with superscript (*) in the same row were significantly different, ** *p* ≤ 0.01.

**Table 4:** Effect of *P. acidilactici* LC-9-1 on serum antioxidant performance of broilers (*n* = 6).

Items	Dietary Treatment		SEM	*p*-Value
	CON	PA		
Day 21				
T-AOC, U/mL	8.36	8.58 *	0.048	0.017
SOD, U/mL	104.85	112.65 *	1.521	0.003
GSH-Px, U/mL	214.83	228.83	2.351	0.088
MDA, nmol/mL	4.78 **	4.34	0.077	0.001
Day 42				
T-AOC, U/mL	9.30	9.40 *	0.022	0.014
SOD, U/mL	129.00	147.50 **	3.610	0.003
GSH-Px, U/mL	242.83	252.50	3.227	0.141
MDA, nmol/mL	5.30	5.17	0.040	0.082

CON = control group, broilers were fed a corn–soybean basal diet, PA = *P. acidilactici* group, broilers were fed a basal diet containing 5.5 × 10^9^ CFU/kg *P. acidilactici* LC-9-1. T-AOC, total anti-oxidant capacity; T-SOD, total superoxide dismutase; GSH-Px, glutathione peroxidase; MDA, malonaldehyde; SEM = standard error of means. The values with superscript (*) in the same row were significantly different, * 0.01 < *p* < 0.05; ** *p* ≤ 0.01.

**Table 5:** Effect of *P. acidilactici* LC-9-1 on histomorphology of the small intestinal sections in broilers (*n* = 6).

Items	Intestine	Treatment		SEM	*p*-Value
		CON	PA		
Day 21					
Jejunum	Villus length, μm	1310.05	1336.47	21.775	0.569
	Crypt depth, μm	182.32	185.83	3.672	0.654
	V/C ratio	7.20	7.25	0.205	0.914
	Epithelial thickness, μm	199.15	213.10	4.415	0.117
Ileum	Villus length, μm	869.15	908.50	16.191	0.241
	Crypt depth, μm	206.20	200.14	4.888	0.561
	V/C ratio	4.24	4.56	0.121	0.205
	Epithelial thickness, μm	219.77	231.23	6.354	0.392
Day 42					
Jejunum	Villus length, μm	1338.93	1478.31	41.007	0.088
	Crypt depth, μm	239.87	234.10	1.749	0.100
	V/C ratio	5.59	6.32	0.198	0.063
	Epithelial thickness, μm	234.83	246.49	6.461	0.392
Ileum	Villus length, μm	842.92	1030.13 **	31.481	<0.001
	Crypt depth, μm	219.55	212.67	3.880	0.401
	V/C ratio	3.86	4.85 **	0.175	<0.001
	Epithelial thickness, μm	349.36	342.28	7.313	0.651

CON = control group, broilers were fed a corn–soybean basal diet, PA = *P. acidilactici* group, broilers were fed a basal diet containing 5.5 × 10^9^ CFU/kg *P. acidilactici* LC-9-1. V/C ratio = villus length (μm)/Crypt depth (μm). SEM = standard error of means. The values with superscript (*) in the same row were significantly different, ** *p* ≤ 0.01.

## Data Availability

The original contributions presented in the study are included in the article/supplementary material, further inquiries can be directed to the corresponding author. The raw data on microbiota of broilers were deposited in NCBI’s Sequence Read Archive (SRA) database and accessible through SRA accession number: PRJNA922579.

## Supplementary Materials

The following supporting information can be downloaded at: https://doi.org/10.5281/zenodo.7510821. Table S1: Analysis composition of basal diets and nutrient level (air-dry basis, %); Table S2: Biochemical characteristics of LC-9-1 based on carbohydrate interpretation using API 50 CHL kit; Table S3: Antibiotic susceptibility profile of potential probiotic *P. acidilactici* LC-9-1; Figure S1: Acid-producing efficiency; Figure S2: Antimicrobial activity.
